# Qualitative versus automatic evaluation of CT perfusion parameters in acute posterior circulation ischaemic stroke

**DOI:** 10.1007/s00234-020-02517-6

**Published:** 2020-08-19

**Authors:** Raffaella Capasso, Stefano Vallone, Nicola Serra, Gabriele Zelent, Luca Verganti, Federico Sacchetti, Guido Bigliardi, Livio Picchetto, Ferdinando Caranci, Andrea Zini

**Affiliations:** 1 Department of Medicine and Health Science “V. Tiberio”, University of Molise, Via Francesco De Sanctis, 1, 86100 Campobasso, Italy; 2 Department of Precision Medicine, School of Medicine, “Luigi Vanvitelli” University of Campania, Naples, Italy; 3 Neuroradiology Unit, Ospedale Civile S.Agostino-Estense, Azienda Ospedaliero-Universitaria di Modena, Dipartimento di Neuroscienze, University of Modena and Reggio Emilia, Modena, Italy; 4 Statistic Unit, Department of Public Health, University of Federico II, Naples, Italy; 5 Stroke Unit, Ospedale Civile S.Agostino-Estense, Azienda Ospedaliero-Universitaria di Modena, Dipartimento di Neuroscienze, University of Modena and Reggio Emilia, Modena, Italy; 6 IRCCS Istituto delle Scienze Neurologiche di Bologna, Department of Neurology and Stroke Center, Maggiore Hospital, Bologna, Italy

**Keywords:** CT perfusion, Posterior ischaemic stroke, Perfusion maps, Automatic, RAPID

## Abstract

**Purpose:**

To compare the diagnostic accuracy (ACC) in the detection of acute posterior circulation strokes between qualitative evaluation of software-generated colour maps and automatic assessment of CT perfusion (CTP) parameters.

**Methods:**

Were retrospectively collected 50 patients suspected of acute posterior circulation stroke who underwent to CTP (GE “Lightspeed”, 64 slices) within 24 h after symptom onset between January 2016 and December 2018. The Posterior circulation-Acute Stroke Prognosis Early CT Score (pc-ASPECTS) was used for quantifying the extent of ischaemic areas on non-contrast (NC)CT and colour-coded maps generated by CTP4 (GE) and RAPID (iSchemia View) software. Final pc-ASPECTS was calculated on follow-up NCCT and/or MRI (Philips Intera 3.0 T or Philips Achieva Ingenia 1.5 T). RAPID software also elaborated automatic quantitative mismatch maps.

**Results:**

By qualitative evaluation of colour-coded maps, MTT-CTP4D and Tmax-RAPID showed the highest sensitivity (SE) (88.6% and 90.9%, respectively) and ACC (84% and 88%, respectively) compared with the other perfusion parameters (CBV, CBF). Baseline NCCT and CBF provided by RAPID quantitative perfusion mismatch maps had the lowest SE (29.6% and 6.8%, respectively) and ACC (38% and 18%, respectively). CBF and Tmax assessment provided by quantitative RAPID perfusion mismatch maps showed significant lower SE and ACC than qualitative evaluation. No significant differences were found between the pc-ASPECTSs assessed on colour-coded MTT and Tmax maps neither between the scores assessed on colour-coded CBV-CTP4D and CBF-RAPID maps.

**Conclusion:**

Qualitative analysis of colour-coded maps resulted more sensitive and accurate in the detection of ischaemic changes than automatic quantitative analysis.

## Introduction

Posterior circulation (PC) stroke accounts for 20–25% of ischaemic strokes and is characterised by a wide range of clinical features that make its clinical diagnosis challenging [1–12]. In emergency setting, dynamic CT perfusion (CTP) has been introduced as a particularly useful technique after non-contrast CT (NCCT) given its easy application, reproducibility of quantitative assessment, prompt availability, low cost and tolerability [13–17]. A mismatch between the irreversibly damaged ischaemic core and the extent of hypoperfused tissue at risk of infarction is an attractive model with which to select ischaemic stroke patients for reperfusion therapies [18–23].

Visual inspection of CTP colour-coded maps can be an effective way to discriminate areas of core infarct and penumbra and may be enough to guide therapeutic choice. It is rapid and simple to use but its strengths may be affected by variability in post-processing, broad range of imaging and computational approaches, selection of parametric maps, expertise skills and the generally qualitative nature of such approaches [23].

On the other side, quantitative CTP analysis has reported to be efficient in demonstrating acute ischaemia, distinguishing salvageable from unsalvageable ischaemic tissue and predicting therapeutic outcome, although protocols and guidelines for quantitative thresholds vary [24–33]. Differences in CTP hardware and software can affect quantified analysis and clearly defined thresholds to manage therapeutic approach have yet to be standardised [24–34]. Thus, expert consensus has underlined the need for standardization in the acquisition, processing and analysis of perfusion imaging [23].

In this context, the recent introduction of a fast, vendor- and operator-independent computational tool working by totally automated lesion segmentation and pixel-wise parametric thresholding for semi-quantitation (RApid processing of PerfusIon and Diffusion [RAPID]) seems to overcome the limits of qualitative approaches to CTP imaging in acute stroke [23, 35–37]. This technological advance is reported to have the potential to improve the standardization and reproducibility of interpretation of perfusion imaging [35].

### Aim of the study

The diagnostic value of CTP in the posterior circulation has been little investigated with few evidences of its reliability and accuracy; moreover, the diagnostic effects of RAPID introduction in the evaluation of infratentorial ischaemic changes have not yet been clarified [3, 4, 38–40]. To the best of our knowledge, these are the first reported instances of PC stroke patient selection using RAPID from European countries.

The aim of this study was to examine and compare the diagnostic accuracy (ACC) in the detection of acute posterior circulation strokes between qualitative evaluation of software-generated colour maps [cerebral blood flow (CBF), cerebral blood volume (CBV), mean transit time (MTT) and time-to maximum (Tmax)] and automatic assessment of CTP data by RAPID software.

## Methods

### Study population and patient selection

According to the approval of the local ethical committee (Area Vasta Emilia Nord, AVEN), imaging data were retrospectively collected from a prospective database of 1498 consecutive patients admitted to the stroke unit of our hospital between January 2016 and December 2018. Written informed consent was waived for this study. All patients were initially evaluated by vascular neurologist in the emergency setting with initiation of an institutional stroke protocol facilitating expedited triage, imaging, interpretation and treatment when appropriate. Patients were enrolled regardless of therapy. The inclusion criteria for this study were (1) suspected acute posterior circulation ischaemic stroke as defined in the Oxfordshire classification [41]; (2) multimodal CT scan dataset including NCCT, supra-aortic CT angiography (CTA) and CTP performed on admission; (3) CT scan performed < 24 h after symptom onset. Exclusion criteria were (1) nondiagnostic image quality, (2) evidence of another cause of neurological deficits (prior stroke with residual deficit, intracranial haemorrhage, tumour etc.), (3) incomplete coverage by the CTP slab of all posterior circulation Acute Stroke Prognosis Early CT Score (pc-ASPECTS) regions and (4) missing follow-up CT or magnetic resonance imaging (MRI).

Out of all patients who underwent multimodal CT due to suspected acute stroke, were selected control patients who fulfilled the same inclusion and exclusion criteria as our case cohort but did not show an acute posterior circulation ischaemia on follow-up CT/MRI scan neither change in at least two perfusion parameters in the same location.

### Imaging protocol

Patients underwent an institutional stroke imaging protocol including NCCT, CTA and CTP at admission. CT protocol was conducted on a 40-mm, 64-detector row clinical system (Lightspeed VCT, GE Healthcare, Milwaukee, Wisconsin). NCCT helical scans were performed from the skull base to the vertex (120 kV, 100–350 auto-mA, 5 mm section thickness). CTAs were conducted from the aortic arch to the top of frontal sinus (120 kV; 200–350 auto-mA; section thickness/interval 0.625/0.375 mm; scan start 6 s after bolus tracking at the level of the ascending aorta) after intravenous administration of 60–70 mL iodinated contrast medium injected at 4 mL/s and followed by 50 mL saline flush.

CTP scans (80 kV, 250 mA and 0.4 s rotation time) consisted of a continuous 65 s acquisition that started 7 s after administration of 50 mL of iodinated contrast medium injected into an antecubital vein at 4 mL/s and followed by 50 mL saline flush; 400 images were reconstructed covering 80 mm. CTP coverage was lowered to cover the cerebellum to the occipital lobes, including all three levels of the pc-ASPECTS.

Follow-up imaging consisted in NCCT. If clinically indicated, MRI was performed either soon after the CTP at the admission or later as follow-up control. MRI exams were achieved using two scanners (Philips Intera 3.0 T and Philips Achieva Ingenia 1.5 T, Philips Medical System, Best, The Netherlands) including diffusion-weighted imaging (DWI) and fluid-attenuated inversion recovery in the transverse plane.

#### CTP post-processing

Raw CTP data were initially processed by a commercially available delay-insensitive deconvolution software [CT Perfusion 4D (CTP4D), GE Healthcare, Waukesha, Milwaukee, Wisconsin]. All patients were obtained CBF, CBV and MTT maps after positioning a region of interest manually in correspondence to a main arterial vessel and a vein. Then, raw CTP data was sent from scanner to a networked computer running fully automated RAPID software (iSchemaView, California) which generated CBF, CBV, MTT, Tmax maps and lesion segmentation and then sent processed images to institutional Picture and Achieving System (PACS). The software also generated a colour perfusion mismatch map for each patient based on the mismatch model Tmax–CBF, providing a comparison between brain regions with substantial reductions in CBF (rCBF < 30%) in pink representing the ischaemic core and regions with significant hypoperfusion in green as reflected by delays in contrast arrival times (Tmax > 6.0 s). The difference between these volumes (mismatch volume) as well as the ratio between these volumes (mismatch ratio) was automatically calculated.

#### NCCT and CTP image analysis

The neuroimaging data was independently reviewed by two board-certified neuroradiologists (with 10 and 4 years of experience in multimodal CT imaging interpretation) blinded to patient clinical information using the institutional PACS; any discrepancies were resolved through a consensus discussion in a separate session.

For NCCT, early ischaemic changes were assessed; an area was considered ischaemic if there was parenchymal hypoattenuation with or without cortical swelling of the brain [42, 43]. Areas of cortical swelling without hypoattenuation were not considered ischaemic [43]. The pc-ASPECTS was used for quantifying the extent of ischaemic areas. Pc-ASPECTS allots 10 points for ischaemic changes assessed by visual inspection [23, 44]. Two points are subtracted in the cases of ischaemic change in any part of pons and midbrain, one point for either side of the cerebellum, one for either side of the thalamus and one for either side of the posterior cerebral artery supplied territory [4, 23, 44, 45]. A pc-ASPECTS of 10 indicates absence of ischaemic changes in the posterior circulation while a pc-ASPECTS of 0 indicates presence of ischaemic changes in all pc-ASPECTS territories [38].

For CTP, a qualitative evaluation of perfusion deficit was initially performed on colour-coded CBV, CBF and MTT maps generated by CTP4D software. Regions with colorimetric asymmetries on CTP maps in comparison with non-affected regions (i.e. contralateral side, different vascular territories) were rated as abnormal. A visible qualitative focal reduction in CBF or CBV and a focal increase MTT on colour-coded maps was rated as abnormal and evaluated with pc-ASPECTS. When a perfusion deficit was suspected in the maps, it was compared with the NCCT scan to exclude false positive caused by beam hardening artefacts, chronic infarct or overlapping with liquoral spaces [12].

In the same way, in a separate scoring session, colour-coded CBF and Tmax maps automatically generated by RAPID software were then visually evaluated assessing pc-ASPECTS for each map. For each patient, the quantitative colour perfusion mismatch map was also evaluated: brain regions with reductions in CBF (< 30%) appeared in pink suggesting ischaemic core while regions with significant increase of Tmax (> 6.0 s) indicating hypoperfusion appeared in green; the abnormal areas identified on perfusion mismatch maps were compared with perfusion changes location on colour-coded maps to assess anatomic consistency in order to exclude artefacts.

PCT images showing no alterations in the same location in at least two visually evaluated perfusion parameter maps neither in automatic maps were considered negative [5].

#### Follow-up imaging evaluation

Standard follow-up imaging consisted in NCCT for all patients which was used to determine the presence of an infarct. Final pc-ASPECTS was calculated on follow-up NCCT and/or on MRI if performed.

Follow-up images were compared with PCT studies for anatomic correlation. The initial NCCT and PCT data was then compared with the follow-up NCCT data or MRI data if available, which was used as criterion standard to calculate sensitivity (SE), specificity (SP) and diagnostic ACC of the abnormalities assessed on NCCT scan and CTP maps for the detection of posterior circulation ischaemic stroke.

### Statistical analysis

For each patient, NCCT and PCT maps showing no ischaemic changes despite follow-up imaging that confirmed the stroke were considered as false negative. PCT maps showing alterations out of the territory of the affected arterial vessel (i.e. contralateral side, anterior circulation) were considered as false positive. NCCT and CTP ischaemic changes were considered true positives if there was an anatomic vascular correspondence to findings on follow-up imaging. NCCT and PCT images showing no alterations in patients whose follow-up imaging did not confirm the stroke were considered as true negative.

Main evaluations were:

- the correspondence between the infarcted area (core) assessed with qualitative evaluation of both colour-coded CTP4D maps and RAPID maps and the ischaemic lesion confirmed on direct follow-up CT or MRI;
- the correspondence among the perfusion changes identified with the qualitative analysis of colour-coded CTP4D maps, colour-coded RAPID maps and the lesions spotted in the RAPID software-generated colour perfusion mismatch map;
- the comparison of the respective ASPECTSs evaluated on NCCT, colour-coded CTP4D maps and colour-coded RAPID maps with the final ASPECTSs assessed on direct follow-up CT or MRI.

The statistical analysis was performed by Matlab statistical toolbox version 2008 (MathWorks, Natick, MA, USA) for Windows at 32 bit. SE, SP and ACC for selected parameters were computed with correspondence confidence intervals (CIs) at 95%. The multiple comparison Cochran’s *Q* tests were used to compare differences of percentages among three or more dependent variables, under the consideration of the null hypothesis that there was no difference between the variables. A value of *p* < 0.05 was considered statistically significant. When the Cochran’s *Q* test was positive (*p* value < 0.05), then a minimum required difference for a significant difference between two proportions was calculated using the minimum required differences method with Bonferroni *p* value corrected for multiple comparisons according to Sheskin. The McNemar’s exact test was used to test the difference between two paired proportions. Additional statistical tests were performed on stroke patient parameters using the Friedman’s ANOVA test. Particularly, if Friedman’s ANOVA test was positive (*p* value <0 .05), a Wilcoxon signed rank post hoc test was performed to individualise significant differences between two diagnostic parameters.

Data are expressed as mean value ± standard deviation (SD) for continuous variables and as absolute number or percentage for categorical variables.

## Results

### Study sample

Among 69 patients meeting inclusion criteria, a total of 50 patients respected the exclusion criteria. Six out of the 50 patients did not show ischaemic core at follow-up imaging neither alteration of at least two perfusion parameters in the same location and were grouped as negative controls. All patients underwent to follow-up NCCT and 28 of them also underwent DWI-MRI. Mean time from multimodal CT study at admission to follow-up CT study and MRI were, respectively, 34.2 **±** 72.6 h and 76.6 **±** 98.8 h. Out of the 28 patients who undergone MRI, 14 patients (50%) underwent DWI study within 8 h after multimodal CT study at admission. Vessel occlusion was located in one of more of the posterior circulation branches involving extracranial vertebral artery in 9 patients, intracranial vertebral artery in 5 patients, basilar artery in 15 patients, posterior cerebral artery in 22 patients and superior cerebellar artery in 7 patients. Inside local stroke protocol multimodal neuroimaging (CT, CTA, CTP or MRI) was used for decision making about inclusion/exclusion criteria for revascularization therapies as intravenous thrombolysis and mechanical thrombectomy. In our study, 26 patients had intravenous thrombolysis, 6 patients underwent to thrombectomy/thromboaspiration, 7 patients had intravenous thrombolysis and underwent to thrombectomy/thromboaspiration while 5 patients were not treated. Negative controls were not treated.

Table 1 provides an overview of demographic features of the study sample and displays the mean ASPECTS evaluated on NCCT at baseline, colour-coded CTP4D maps, colour-coded RAPID maps, follow-up NCCT and DWI-MRI.

**Table 1:** Demographic features and mean ASPECTSs evaluated on respective images of all 50 patients included in the study

	Stroke patients		Negative controls	
	Nr./percentage	Mean ± SD	Nr./percentage	Mean ± SD
Nr. patients	44	—	6	—
Age	—	71.09 ± 15.64	—	69 ± 9.52
Gender (male)	(30/44)/68.18%	—	(4/6)/66.67%	—
Baseline NCCT	—	ASPECTS	—	ASPECTS
		9.48 ± 1.12		10.0 ± 0.0
MTT (CT perfusion 4D)	—	6.80 ± 2.84	—	9.17 ± 0.90
CBV (CT perfusion 4D)	—	8.16 ± 2.40	—	10.0 ± 0.0
CBF (CT perfusion 4D)	—	7.52 ± 2.61	—	10.0 ± 0.0
Tmax (colour-coded rapid)	—	6.41 ± 3.06	—	9.33 ± 0.94
CBF (colour-coded rapid)	—	8.16 ± 2.33	—	10.0 ± 0.0
follow-up NCCT	—	6.98 ± 2.51(44 patients)	—	10.0 ± 0.0
Final ASPECTS	—	6.93 ± 2.35(44 patients)	—	10.0 ± 0.0
MR-DWI	—	7.04 ± 2.14(28 patients)	—	—
Follow-up NCCT	—	6.93 ± 2.69(16 patients)	—	10.0 ± 0.0

### Study sample

### Diagnostic accuracy

Table 2 displays SE, SP, and ACC relative to baseline NCCT, visually evaluated colour-coded MTT, CBV and CBF maps generated by CTP4D software, visually evaluated colour-coded Tmax and CBF maps generated by RAPID software, automatic quantitative Tmax and CBF parameters spotted in the RAPID software-generated colour perfusion mismatch map, using follow-up CT or MRI as criterion standard.

**Table 2:** Sensitivity, specificity, diagnostic accuracy, false negative and false positive cases relative to baseline NCCT, visually evaluated colour-coded MTT, CBV and CBF maps generated by CT Perfusion 4D software, visually evaluated colour-coded Tmax and CBF maps generated by RAPID software (Tmax^1^ and CBF^1^, respectively), automatic quantitative Tmax and CBF parameters spotted in the RAPID software-generated colour perfusion mismatch map (Tmax^2^ and CBF^2^, respectively)

Parameters	Sensitivity (CI at 95%)	Specificity (CI at 95%)	Accuracy (CI at 95%)	False negative	False positive
Baseline NCCT	29.6% (13/44) (16.8–45.2%)	100% (6/6) (54.1–100%)	38% (19/50) (25–52.7%)	31/44 70.4%	-
MTT (CT perfusion 4D)	88.6% (39/44) (75.4–96.2%)	50% (3/6) (11.8–88.2%)	84% (42/50) (70.3–92.8%)	5/44 11.4%	3/6 50%
CBV (CT perfusion 4D)	63.6% (28/44) (47.8–77.6%)	100% (6/6) (54.1–100%)	68% (34/50) (53.2–80.3%)	16/44 36.4%	-
CBF (CT perfusion 4D)	81.8% (36/44) (67.3–91.8%)	100% (6/6) (54.1–100%)	84% (42/50) (70.3–92.8%)	8/44 18.2%	-
Tmax^1^ (colour-coded rapid)	90.9% (40/44) (78.3–97.5%)	66.7% (4/6) (22.3–95.7%)	88% (44/50) (75–95.6%)	4/44 9.1%	2/6 33.3%
CBF^1^ (colour-coded rapid)	61.4% (27/44) (45.5–75.6%)	100% (6/6) (54.1–100%)	66% (33/50) (51.1–78.6%)	17/44 38.6%	-
Tmax^2^ (quantitative rapid)	65.9% (29/44) (50.1–79.5%)	100% (6/6) (54.1–100%)	70% (35/50) (55.2–81.9%)	11/44 25%	4/6 66.7%
CBF^2^ (quantitative Rapid)	6.8% (3/44) (1.4–18.7%)	100% (6/6) (54.1–100%)	18% (9/50) (9.1–31.5%)	41/44 93.2%	-

For each patient, follow-up CT or MRI was used as criterion standard. Confidence interval (CI) at 95%

### Diagnostic accuracy

SE, SP and diagnostic ACC of qualitative evaluation on baseline NCCT, colour-coded CTP4D maps (MTT, CBV and CBF), colour-coded Tmax and CBF RAPID maps (Tmax^1^ and CBF^1^), and automatic quantitative Tmax and CBF RAPID software-generated perfusion mismatch map (Tmax^2^ and CBF^2^), were compared.

**Fig. 1:**
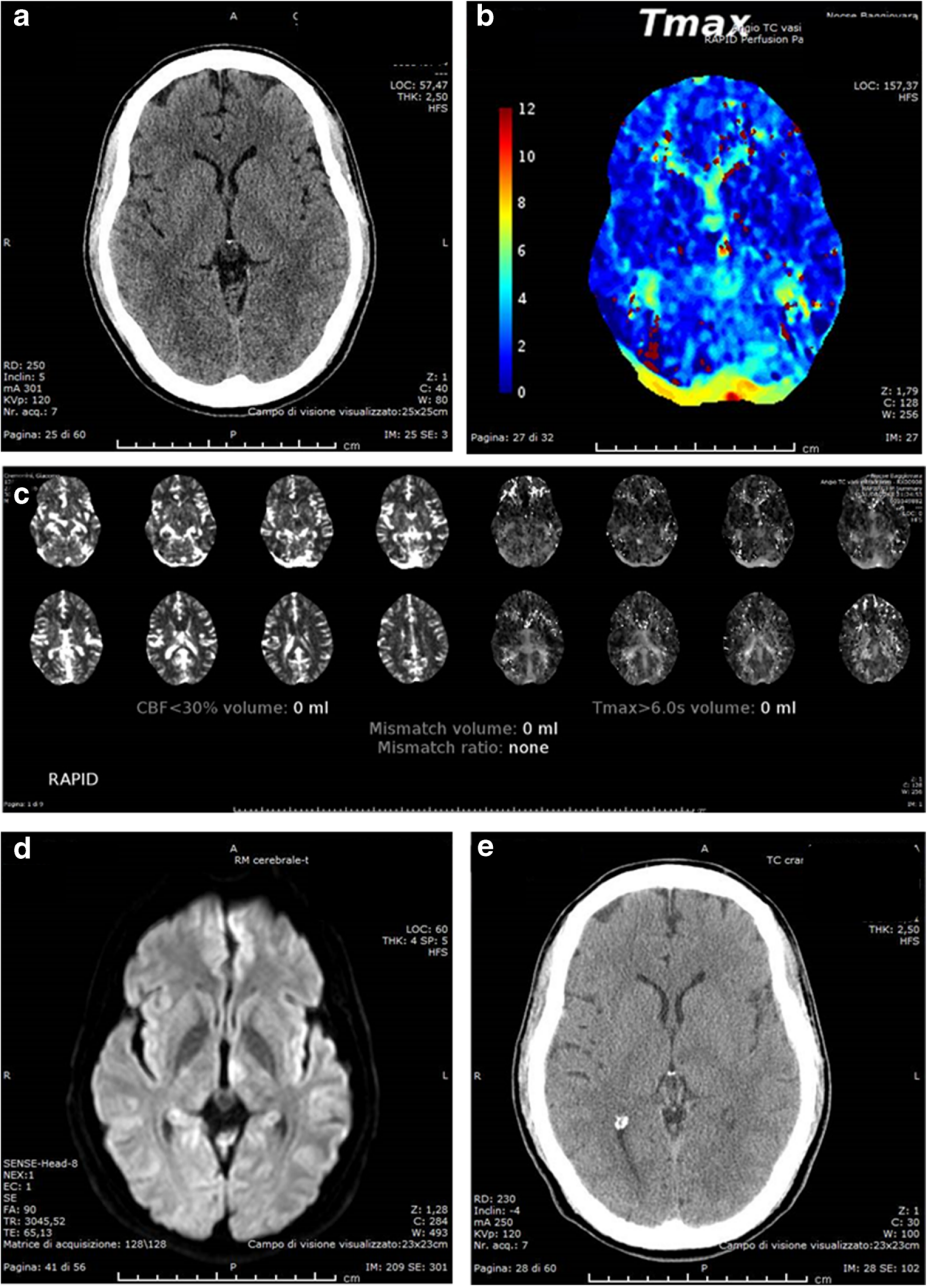
Left thalamic lacunar infarct: **a** ASPECTS 10 on initial NCCT, **b** RAPID colour-coded Tmax map shows perfusion alteration at left thalamic level but not displayed on RAPID automatic mismatch map (**c**); the lesion was confirmed on DWI (**d**) and also visible on follow-up CT (**e**)

**Fig. 2:**
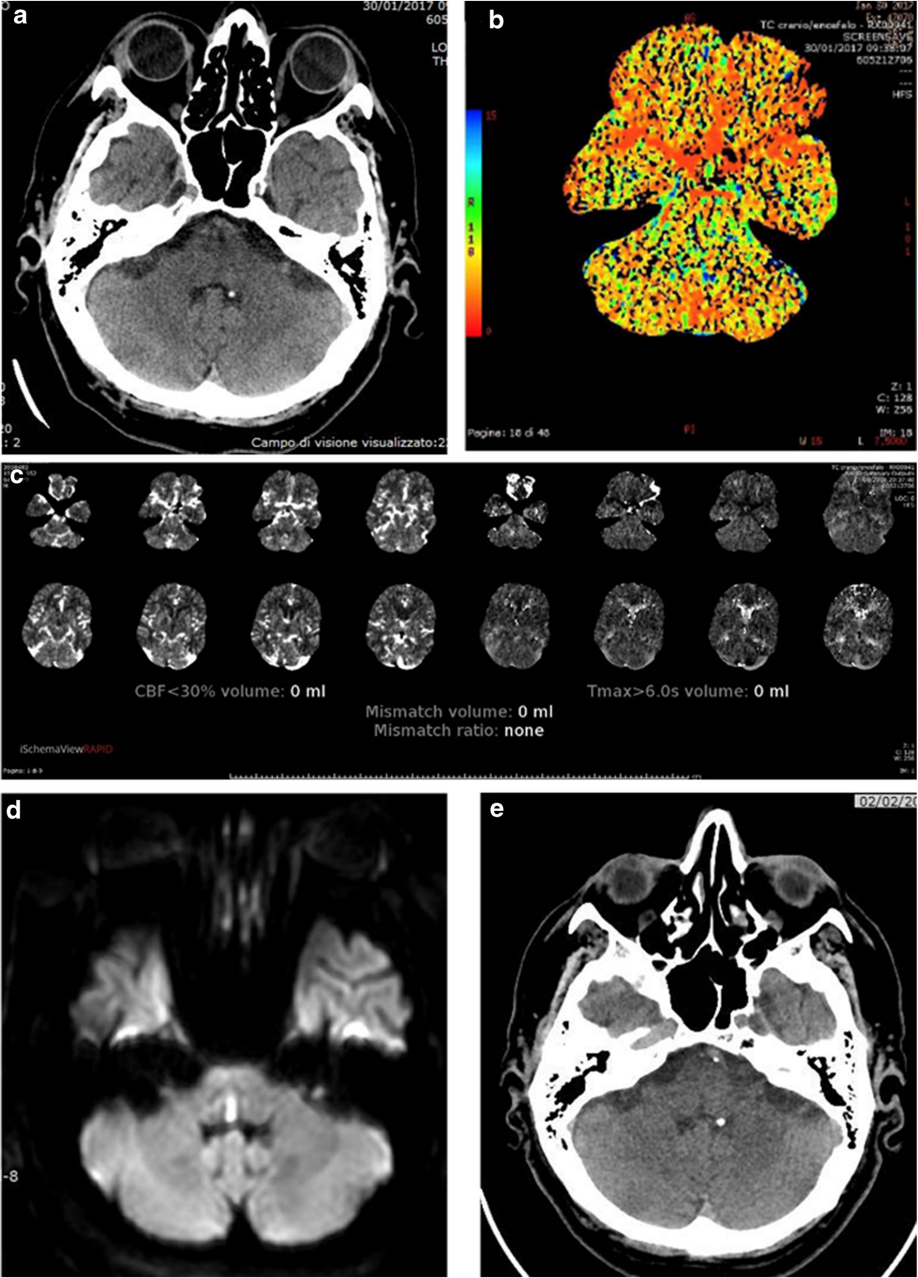
Mesencephalic lacunar infarct not clearly identified on initial NCCT (**a**), neither on colour-coded MTT (**b**) map and RAPID automatic mismatch map (**c**) but detected by DWI (**d**) and confirmed on follow-up CT (**e**)

The sensitivity of qualitative evaluation of colour-coded MTT map and colour-coded Tmax^1^ map resulted significantly higher than the other ones (MTT: 88.6%, *p* < 0.05; Tmax^1^: 90.9%, *p* < 0.05), while NCCT at baseline and CBF^2^ provided by RAPID quantitative perfusion mismatch maps had the lowest values (29.6% and 6.8% *p* < 0.05, respectively). NCCT at baseline and CBF^2^ provided by RAPID quantitative perfusion mismatch maps also showed false negative cases in most patients variously located in one or more pc-ASPECTS territories. Automatic Tmax^2^ evaluation provided by RAPID overlooked 11 ischaemic lesions which mostly (8/11) were lacunar infarcts involving thalamus (4/8) or brainstem (4/8) while the remaining were larger lesions involving occipital lobe an cerebellum (3/11) (Fig. 1). Visual qualitative evaluation of MTT overlooked ischaemic changes in five patients located in pc-ASPECT pons-midbrain region in five cases associated to thalamus involvement in two cases, visual qualitative evaluation of Tmax^1^ overlooked ischaemic changes in four patients located in pc-ASPECT pons-midbrain regions in three cases and thalamus in one case (Fig. 2). No significant difference was found between MTT map and Tmax^1^ map sensitivities (88.6% < 90.9%, *p* > 0.05). For CBV and CBF parameters, the number of false negative cases located in one or more pc-ASPECTS regions probably reflected the potentially salvageable tissue. However, it is relevant that automatic assessment of CBF^2^ provided by RAPID mismatch map resulted negative for core extent in most (41/44) of patients, among them 20 were lacunar infarcts involving thalamus (15/20) and brainstem (5/20).

About specificity, there was a significant difference among parameters but post hoc test did not confirm significant differences considering all pairwise comparison. However, qualitative evaluation of MTT and Tmax^1^ maps and automatic evaluation of Tmax^2^ provided by RAPID quantitative perfusion mismatch maps were characterised by some false positive cases showing perfusion disturbance in anterior circulation territory.

About diagnostic accuracy, MTT and Tmax^1^ showed the highest values (84% and 88% *p* < 0.05, respectively) while CT at baseline and CBF^2^ provided by RAPID quantitative perfusion mismatch maps had the lowest values (38% and 18% *p* < 0.05, respectively). Between MTT and Tmax^1^ map, there was no significant difference (88% > 84%, *p* > 0.05) in terms of diagnostic accuracy.

In addition, sensitivity, specificity and diagnostic accuracy were compared among CBF, CBF^1^ and CBF^2^ parameters (respectively, qualitatively evaluated on colour-coded CTP4D, colour-coded RAPID maps and automatically provided by RAPID quantitative perfusion mismatch maps) and between Tmax^1^ and Tmax^2^ parameters (respectively, qualitatively evaluated on colour-coded CTP4D and automatically provided by RAPID quantitative perfusion mismatch maps) (Table 3).

**Table 3:** Comparison among CBF parameters and Tmax parameters evaluated in this study in terms of sensitivity, specificity and diagnostic accuracy

Parameters	CBF (colour-coded CT perfusion 4D)	CBF^1^ (colour-coded rapid)	CBF^2^ (quantitative RAPID)	Statistical test
Sensitivity	81.8% (36/44)	61.4% (27/44)	6.8% (3/44)	*p* < 0.001 (Q)
Specificity	100% (6/6)	100% (6/6)	100% (6/6)	*p* > 0.05 (Q)
Diagnostic Accuracy	84% (42/50)	66% (33/50)	18% (9/50)	*p* < 0.001 (Q)
Parameters		Tmax^1^ (colour-coded rapid)	Tmax^2^ (quantitative RAPID)	Statistical test
Sensitivity		90.9% (40/44)	65.9% (29/44)	90.9% > 65.9%, *p* = 0.001 (M)
Specificity		69.7% (4/6)	100% (6/6)	69.7.9% < 100%, *p* = 0.5 (M)
Diagnostic Accuracy		88% (44/50)	70% (35/50)	88% > 70%, *p* = 0.0225 (M)

*Q* Cochran’s *Q* test, *M* McNemar exact test

CBF^2^ assessment provided by quantitative RAPID perfusion mismatch maps showed significant lowest sensitivity (6.8%) in comparison with qualitative evaluations of both colour-coded CBF-CTP4D and CBF^**1**^-RAPID maps (81.8% and 61.4%, respectively); no significant difference of sensitivity was found between qualitative evaluations of colour-coded CBF-CTP4D and CBF^1^-RAPID maps (81.8% > 61.4%, *p* > 0.05). Analogous results were observed for diagnostic accuracy, while there were no significant differences of specificity among CBF, CBF^1^ and CBF^2^ parameters (respectively, qualitatively evaluated on colour-coded CTP4D and RAPID maps and automatically provided by RAPID quantitative perfusion mismatch maps).

Qualitative evaluation of colour-coded Tmax^1^-RAPID maps showed significant higher sensitivity and accuracy than quantitative assessment of Tmax^2^ automatically provided by RAPID perfusion mismatch maps (90.9% > 65.9% and 88% > 70%, respectively) while about specificity, there were no significant difference (69.7.9% < 100%, *p* = 0.5).

### Comparison between qualitative and automatic quantitative perfusion parameter evaluations

## PC-ASPECTS

Interobserver agreement for visual evaluation and ASPECT scoring of MTT, CBV, CBF, Tmax^1^ and CBF^1^ maps was established by the exact count approach: MTT, 0.82; CBV, 0.88; CBF, 0.86; Tmax^1^, 0.84; CBF^1^, 0.8.

Pc-ASPECTSs assessed for each qualitative imaging evaluation [NCCT at baseline, visually evaluated colour-coded MTT, CBV and CBF maps generated by CTP4D software, visually evaluated colour-coded Tmax^1^ and CBF^1^ maps generated by RAPID software and the reference standard imaging (follow-up NCCT or MRI)] in stroke patients were analysed by the Friedman’s ANOVA test and pairwise Wilcoxon signed-rank post hoc test (Table 4). No significant differences were revealed among values corresponding to the reference standard (2.82), MTT (colour-coded CTP4D, 2.80) and Tmax^1^ (colour-coded Rapid, 2.48) neither between MTT (colour-coded CTP4D) and Tmax^1^ (colour-coded Rapid). No significant differences were even found between CBV (colour-coded CTP4D, 5.14) and CBF^1^ (colour-coded Rapid, 4.90) values. NCCT at baseline, instead, significantly differed from all the other parameters.

**Table 4:** Pairwise comparison with Wilcoxon test after positive Friedman’s ANOVA test; for each tested variable (left column), the other variables from which (right column) it significantly differed are reported

Variables	Mean rank	Significant differences (*p* < 0.05)
(1) Reference standard imaging	2.82	NCCT at baseline, CBV (colour-coded CT Perfusion 4D), CBF (colour-coded CT Perfusion 4D), CBF^1^ (colour-coded rapid)
(2) NCCT at baseline	6.08	All other variables
(3) MTT (colour-coded CT perfusion 4D)	2.80	NCCT at baseline, CBV (colour-coded CT perfusion 4D), CBF (colour-coded CT perfusion 4D), CBF^1^ (colour-coded rapid)
(4) CBV (colour-coded CT perfusion 4D)	5.14	Reference standard imaging, NCCT at baseline, MTT (colour-coded CT perfusion 4D), CBF (colour-coded CT perfusion 4D), Tmax^1^ (colour-coded rapid)
(5) CBF (colour-coded CT perfusion 4D)	3.80	All other variables
(6) Tmax^1^ (colour-coded rapid)	2.48	NCCT at baseline, CBV (colour-coded CT Perfusion 4D), CBF (colour-coded CT Perfusion 4D), CBF^1^ (colour-coded rapid)
(7) CBF^1^ (colour-coded rapid)	4.90	Reference standard imaging, NCCT at baseline, MTT (colour-coded CT perfusion 4D), CBF (colour-coded CT perfusion 4D), Tmax^1^ (colour-coded rapid)

Furthermore, ASPECTSs assessed by qualitative evaluations were compared with the final score assigned by the reference standard imaging (control NCCT or MRI), distinguishing if the qualitative score was equal, higher and lower than the reference standard one. The comparison is resumed in Table 5.

Baseline NCCT showed significant most frequent higher values (84%, *p* < 0.05) and less frequent equal values (16%, *p* < 0.05). MTT (colour-coded CT perfusion 4D) and Tmax^1^ (colour-coded rapid) were the parameters with significant less frequent higher values (24%, *p* < 0.05; 22%, *p* < 0.05, respectively) and more frequent lower values (32%, and 38% *p* < 0.05, respectively) in comparison with the others; MTT (colour-coded CTP4D) and Tmax^1^ (colour-coded rapid) parameters resulted statistically equivalent to individualise perfusion changes.

**Table 5:** Comparison among the ASPECTSs assessed by qualitative evaluations with the final score assigned by the reference standard imaging

Parameters	% higher	% lower	% equal
NCCT at baseline	84% (42/50)	0.0% (0/50)	16% (8/50)
MTT (colour-coded CT perfusion 4D)	24% (12/50)	32% (16/50)	44% (22/50)
CBV (colour-coded CT perfusion 4D)	68% (34/50)	10% (5/50)	22% (11/50)
CBF (colour-coded CT perfusion 4D)	42% (21/50)	14% (7/50)	44% (22/50)
CBF^1^(colour-coded rapid)	60% (30/50)	8% (4/50)	32% (16/50)
Tmax^1^ (colour-coded rapid)	22% (11/50)	38% (19/50)	40% (20/50)
Statistical test	*p* < 0.001 (Q) NCCT, *p* < 0.05 (Sh) MTT, *p* < 0.05 (Sh) Tmax^1^, *p* < 0.05 (Sh)	*p* < 0.001 (Q) MTT, *p* < 0.05 (Sh) Tmax^1^, *p* < 0.05 (Sh)	*p* < 0.001 (Q) NCCT, *p* < 0.05 (Sh)

*Q* Cochran’s *Q* test, *Sh* Sheskin’s procedure

Pc-ASPCETS comparison with the final score evaluated on the reference standard imaging

### Pairwise comparison

## Discussion

### Ischaemic lesion detection

Brain imaging plays a key role in the evaluation of patients suspected of ischaemic stroke allowing to reach a confident diagnosis of infratentorial stroke [5, 12]. MRI with DWI represents the diagnostic gold standard for the detection of ischaemic lesions in the PC territories [4, 5]. However, in many institutions, CT is more readily accessible in the acute phase and less time consuming than MRI being also helpful if MRI is contraindicated or unavailable [1, 4, 5]. CTP has gained widespread application thanks to its accessibility, proved higher sensitivity and accuracy than NCCT alone in detecting acute ischaemic changes also in PC territories [3, 5, 12, 17].

While the infarcted core typically shows a uniform decrease in CBF and CBV, the penumbra is characterised by a reduction of the CBF value with a normal or even increased CBV due to the vasodilation of the precapillary arteries and venous obstruction [12, 19]. Such areas can also be characterised by prolonged MTT extending beyond areas of core infarct and have been called CBV/MTT mismatch [20]. According to recent evidence, relative cerebral blood flow (rCBF) seems to better perform than other parameters, including CBV, in predicting the infarct core, and Tmax is reported to more accurately measure the penumbra in patients with acute ischaemic stroke: CBF/Tmax mismatch [18, 19, 23].

Prior studies have shown that ischaemic core presents > 70% reduction in CBF (rCBF < 0.3) in comparison with the mean CBF of normally perfused brain parenchyma while a Tmax delay of > 6 s is a good predictor of critically hypoperfused tissue that is at risk to progress to infarction in the absence of timely reperfusion; RAPID mismatch maps in our study were automatically elaborated according to these thresholds [18, 23, 28–33].

Concordant to other studies, our findings confirmed that CTP in PC allows to detect significantly more ischaemic strokes than NCCT [4, 5, 39]. As NCCT presented low SE and ACC values, the addiction of CTP in the diagnostic work-up in our patients suspected of an ischaemic posterior circulation stroke significantly increased diagnostic ACC.

### MTT and Tmax

Perfusion change in the posterior circulation has previously been described to be most frequent and pronounced on MTT and Tmax maps [4, 5, 38]. In agreement with other studies, MTT and Tmax visually assessed on colour-coded maps (respectively, generated by CTP4D and RAPID software) in our study were the most significant sensitive and accurate parameters in the detection of PC ischaemic lesions [5]; furthermore, they showed no significant differences in terms of sensitivity (88.6% < 90.9%, *p* > 0.05) and ACC (88% > 84%, *p* > 0.05) despite a superiority of Tmax as described in literature [29].

Automatic quantitative assessment of Tmax^2^ (provided by RAPID perfusion mismatch maps) presented significant lower sensitivity and accuracy compared with qualitative visual evaluation of Tmax^1^ maps. Automatic Tmax^2^ evaluation, qualitative evaluation of MTT and Tmax^1^ overlooked ischaemic changes mainly located in pons-midbrain regions and thalamus.

Despite potential shortcomings due to beam hardening artefacts, previous studies investigating the specificity of CTP in the detection of posterior circulation infarcts demonstrated specificity higher than 90% [5, 17]. In our study, CTP parameters presented different specificity values (ranging from 50% up to 100%) but post hoc test revealed no significant differences of specificity among all assessed CTP parameters, and this result was probably due to small number of control patients considered in this study. As expected, CBF resulted more specific than MTT for stroke because MTT values can be prolonged in transitory ischaemic attack as well as stroke [19]. As previously described in literature, we found that qualitatively evaluated MTT and Tmax^1^ had low specificity for perfusion deficits [12].

### CBV and CBF

The detection of the ischaemic core is most relevant for the prediction of the functional outcome of stroke patients and core extent is described to be better delineated on CBF compared with CBV maps because CBV parameter may underestimate core extent [21, 38].

In agreement with these evidences, in our study, qualitative evaluation of CTP4D maps showed lower sensitivity and accuracy of CBV parameter compared with CBF ones although without significant difference (63.6 < 81.8%, *p* > 0.05).

CTP maps, especially CBF abnormalities, are described to significantly differ among commercial software even when using identical source data [34]; however, we found that visually evaluated CBF and CBF^1^ maps—respectively, generated both by CTP4D and RAPID software—presented no significant difference of sensitivity (81.8% > 61.4%, *p* > 0.05) and accuracy (6.8% < 18%, *p* > 0.05). Instead, the automatic quantitative assessment of CBF^2^ (provided by RAPID perfusion mismatch maps) presented significant lower sensitivity and accuracy as compared with qualitative visual evaluation of both CTP4D and RAPID colour-coded CBF maps. CBV, CBF, CBF^1^ and CBF^2^ parameters presented the highest specificity among all assessed CTP parameters without false-positive cases; this is a critical point to not erroneously exclude patients from therapeutic intervention.

**Fig. 3:**
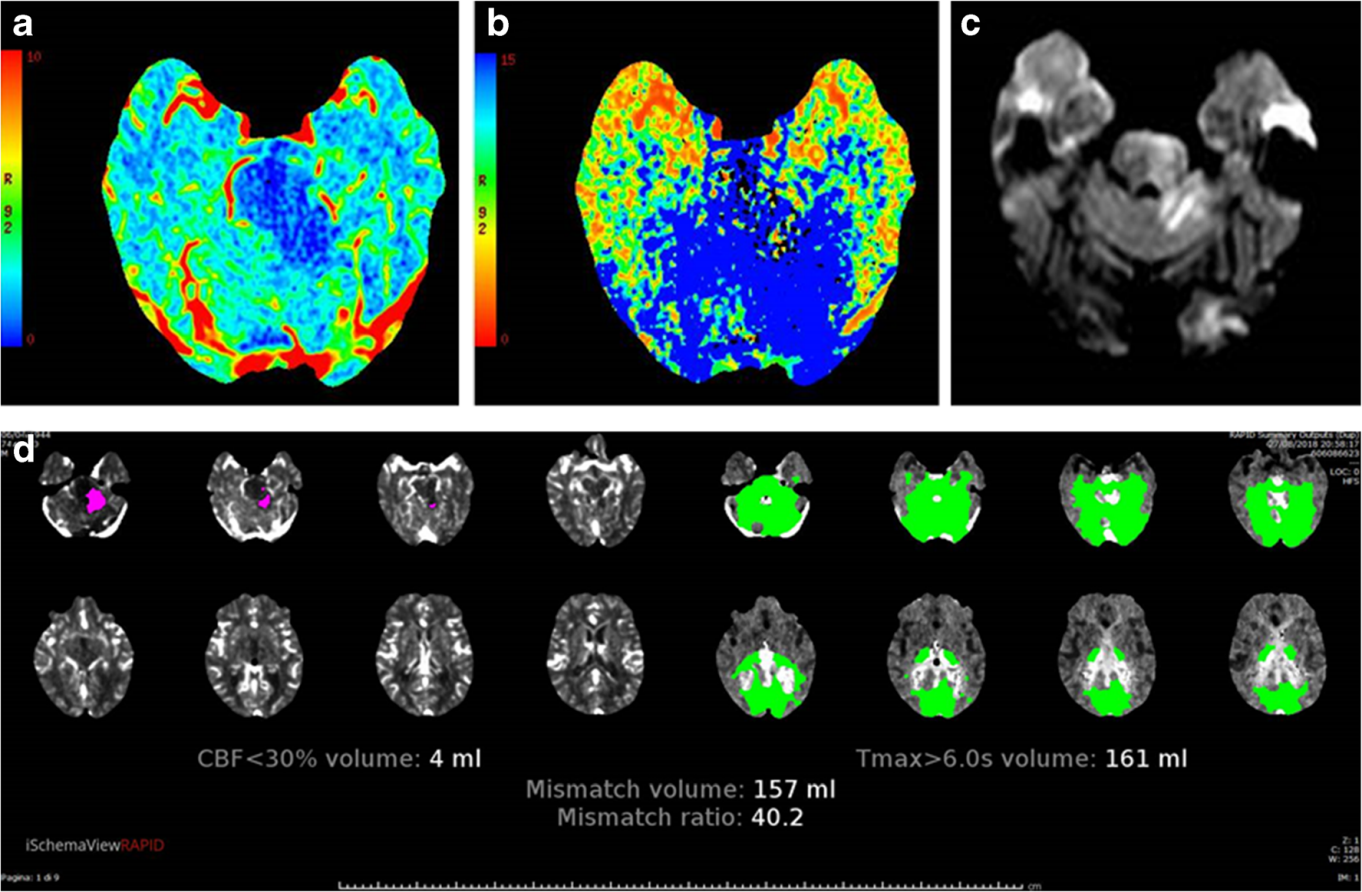
Left cerebellar and mesencephalic ischaemic changes recognised as core and penumbra, respectively, on CBV (**a**) and MTT (**b**) colour-coded CT perfusion 4D maps; the presence of ischaemic core was confirmed by DWI-MRI performed immediately after to better evaluate the involvement of mesencephalon; the automatic RAPID mismatch map (**d**) accurately depicted the extent of ischaemic core and penumbra

**Fig. 4:**
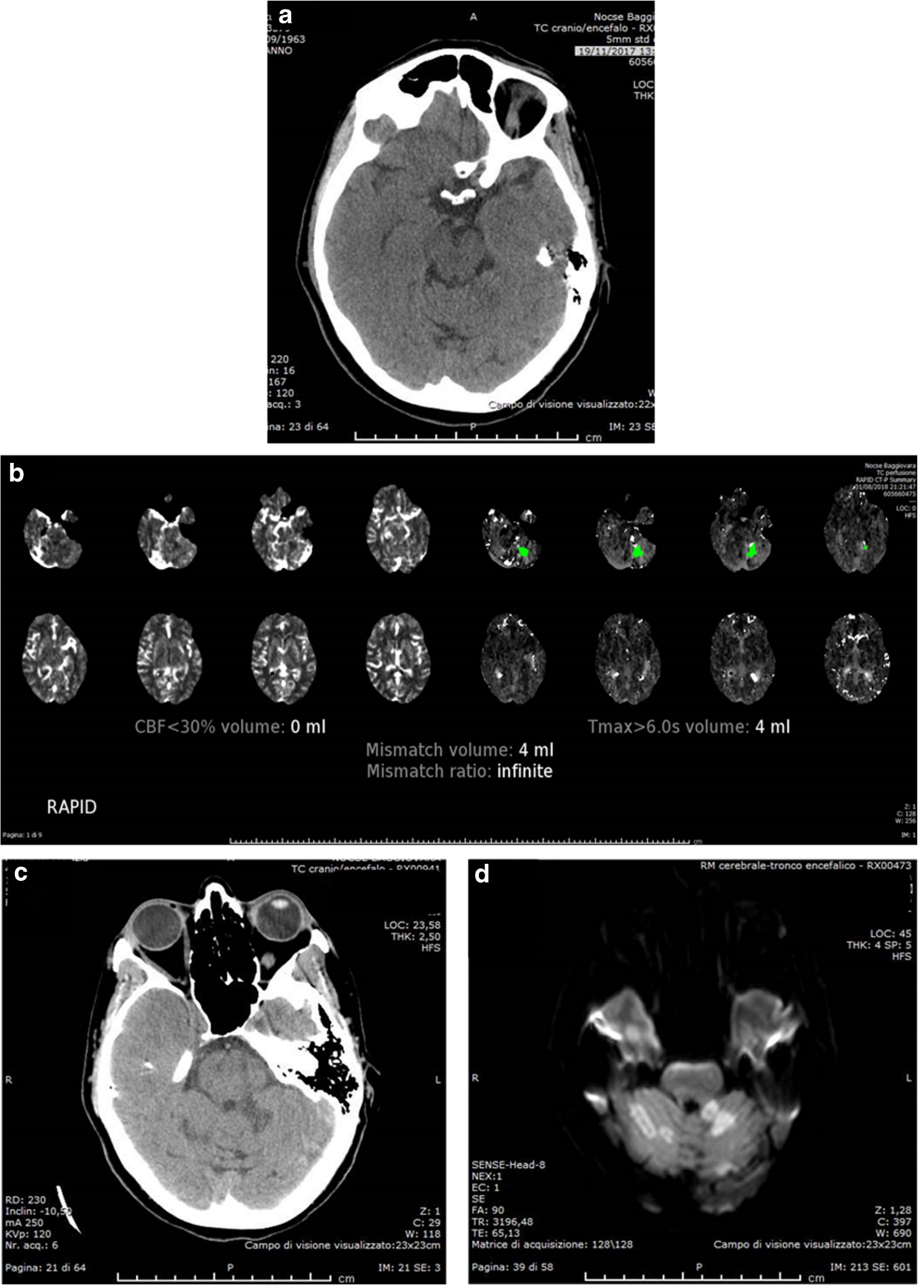
Left small cerebellar ischaemic lesion visible on initial NCCT (**a**) as hypodensity, not recognised as ischaemic core by the automatic RAPID mismatch map (**b**) but confirmed on follow-up CT (**c**) and DWI (**d**)

Despite other reports using the mismatch model have suggested that visual assessment is unreliable and that automatic processing of CTP can provide an operator-independent mismatch classification improving standardization and reproducibility of interpretation (Fig. 3), in our study, RAPID perfusion mismatch maps failed to adequately reveal the ischaemic core extent in the major part of the patients (41/44) as compared with the qualitative analysis [27]. A relevant result of our study was that CBF^2^ evaluation (rCBF automatically provided by RAPID quantitative perfusion mismatch map) was characterised by significant low sensitivity and accuracy values resulting less adequate even than NCCT for detecting ischaemic lesions in PC regions. Indeed, automatic RAPID assessment did not identify ischaemic lesions visually recognised on NCCT in 10 patients (thalamic lacunar infarct in 5 patients and small cerebellar infarct in 3 patients) (Fig. 4). Only in one patient, automatic RAPID mismatch map depicted an ischaemic core (in the left cerebellar hemisphere) not identified on NCCT.

## PC-ASPECTS

The determination of pc-ASPECTs on colour-coded perfusion maps does not require any volumetric software and can be applied to NCCT as well to colour-coded perfusion maps [45].

In our study, the pc-ASPECTSs assessed by NCCT were mainly higher and significantly different from the corresponding final pc-ASPECTs evaluated on control CT/MRI scan and NCCT presented the lowest rate of scores equal to the final ones. These findings are clearly related to the low SE of NCCT which can depict only the ischaemic core and not perfusion changes at risk of infarction.

We found no significant differences among the scores assessed on control CT/MRI scan, colour-coded MTT and Tmax^1^ maps resulting MTT and Tmax^1^ the most efficient parameters to evaluate the patients’ status. This finding can be interpreted as the result of the progression of ischaemic hypoperfusion to necrosis in most of our patients. Moreover, MTT and Tmax^1^ maps led to assign lower ASPECTS significantly more (32%, *p* < 0.05; 38%, *p* < 0.05, respectively) and higher ASPECTS significantly less (24%, *p* < 0.05; 22%, *p* < 0.05, respectively) frequently than the other parameters; a lower score than final one reflected the presence of hypoperfusion not progressed to necrosis while a higher score than final one can be interpreted as a worsening of ischaemic damage and/or as an overlooked ischaemic change. In four patients with midbrain infarct, all perfusion colour-coded maps were scored 10 and the automatic quantitative assessment did not identify these lesions.

Interesting was that no significant differences were found between the scores assessed on colour-coded MTT and Tmax^1^ maps neither between the scores assessed on colour-coded CBV and CBF^1^ maps; according to this, in our study, the mismatch models MTT-CBV and Tmax^1^-CBF could be considered comparable being not different the determination of hypoperfusion by MTT and Tmax^1^ neither different the assessment of core extent by CBV and CBF^1^.

### Limits

A possible limitation of this study is the retrospective design. Intravenous thrombolysis and thrombectomy may also have influenced SE and SP of perfusion maps because they can lead to temporary reperfusion in the potentially salvageable penumbra. Furthermore, the use of 64-slice CT did not allow the study of the whole parenchyma and a careful positioning of the slab was necessary although resulting not always adequate. This could have influenced the SE of CTP maps.

## Conclusion

In this study, CTP showed good diagnostic accuracy in the identification of acute vascular ischaemic lesions of the PC and the infarct detection of CTP was significantly higher than NCCT [12, 39]. The most sensitive perfusion parameters were MTT and Tmax. However, sensitivity increases with infarct size being the draw-back of CTP in detection of small-volume infarctions [5]. Indeed, detection of ischaemic lesion in the brainstem remains challenging due to beam hardening artefacts [5, 45].

Independently to the software employed, qualitative analysis of colour-coded maps resulted more sensitive in the detection of ischaemic changes than automatic quantitative analysis. RAPID software-generated mismatch maps overlooked and underestimated the extent of the ischaemic core in the major part of the patients as compared with the qualitative analysis [12]. In our study, the limits of identification of the lesions by automatic quantitative mismatch maps mainly lied in the thalamus and brainstem. Visual assessment of CTP pc-ASPECTS on colour-coded perfusion maps revealed comparable scores between MTT and Tmax^1^ as well as between CBV and CBF^1^ suggesting the equivalence of both mismatch models (MTT-CBV and Tmax-CBF) commonly applied in acute setting with implications for treatment decision-making.

For these reasons, the multimodal evaluation of the whole neuroradiological examination (NCCT, all qualitative and quantitative CTP maps) must be underlined [46].

Given the advantages of a more rapid and operator independence elaboration of perfusion maps and their easier interpretation, this opens the potential for automatic software implementation and optimization of perfusion parameters’ thresholds for the evaluation of posterior circulation ischaemia [12, 35].

## Data Availability

The data that support the findings of this study are available on request from the corresponding author [R.C.].
